# Gliadel use in a pregnant patient with malignant glioma: a case report

**DOI:** 10.3389/fonc.2025.1700845

**Published:** 2025-10-31

**Authors:** Vishnu Venkitasubramony, Meghan Tahbaz, Kian A. Huang, Mary A. Cain, Charles Preuss, Nam D. Tran

**Affiliations:** ^1^ Morsani College of Medicine, University of South Florida, Tampa, FL, United States; ^2^ Dept of Neurology, Cedars-Sinai, Los Angeles, CA, United States; ^3^ Dept of Neuro-Oncology, Moffitt Cancer Center and Research Institute, Tampa, FL, United States

**Keywords:** pregnancy, Gliadel wafers, carmustine, case report, malignant glioma

## Abstract

Malignant gliomas during pregnancy represent a rare and complex clinical challenge, with limited data to guide management. We report a case of a 35-year-old woman with World Health Organization (WHO) grade III anaplastic astrocytoma who presents at 18 weeks’ pregnancy with progressive disease. She underwent craniotomy for tumor resection with implantation of intralesional carmustine (Gliadel) wafers. Histopathology confirmed progression to WHO grade IV astrocytoma. The patient remained neurologically stable and delivered a healthy infant by cesarean section at 26 weeks’ gestation. Postpartum, she initiated systemic therapy but ultimately experienced progression and died 4 months later. Her daughter remains healthy with normal development more than 14 years after birth. Carmustine use during pregnancy is rarely reported and intralesional carmustine use in pregnancy has never been reported. Pharmacokinetics suggests minimal systemic absorption, limiting fetal exposure. Our case adds to the limited literature, highlighting the feasibility of local chemotherapy with carmustine wafers during the second trimester.

## Introduction

The annual incidence of primary malignant brain tumors in women in the United States is 2.6 for every 100,000, with gliomas being the most prevalent histological type (1). Cancer during pregnancy occurs in approximately 1 in 1,000 pregnancies (0.07% to 0.1% of all malignancies) (2). The tumor’s natural history is altered by hormonal, vascular, and immunologic changes that may accelerate growth rates and exacerbate cerebral edema. In a multi-institutional retrospective study, Peeters et al. (3) identified an increase in tumor growth rates during pregnancy in 87% of cases. Furthermore, clinical deterioration occurred in 38% of cases. The concurrence of pregnancy and glioma is therefore rare, and management is complicated by the competing priorities of maternal treatment and fetal safety. Herein, we report the case of a pregnant patient with grade IV glioblastoma who was treated with intralesional carmustine (Gliadel) chemotherapy during pregnancy.

## Case presentation

A 35-year-old primipara patient at 18 weeks’ gestation presented with neurologic decline, including aphasia, right hemiparesis, and seizures. She was initially diagnosed with an astrocytoma in 2001 and treated surgically with gross total resection. A recurrence in 2002 revealed progression to World Health Organization (WHO) grade III anaplastic astrocytoma and was managed with surgery followed by concurrent chemoradiation with temozolomide. She only completed 9 out of 12 cycles of maintenance temozolomide due to chemotherapy-induced thrombocytopenia. Family history, social history, and relevant genetics were non-contributory to this case.

**Figure 1:**
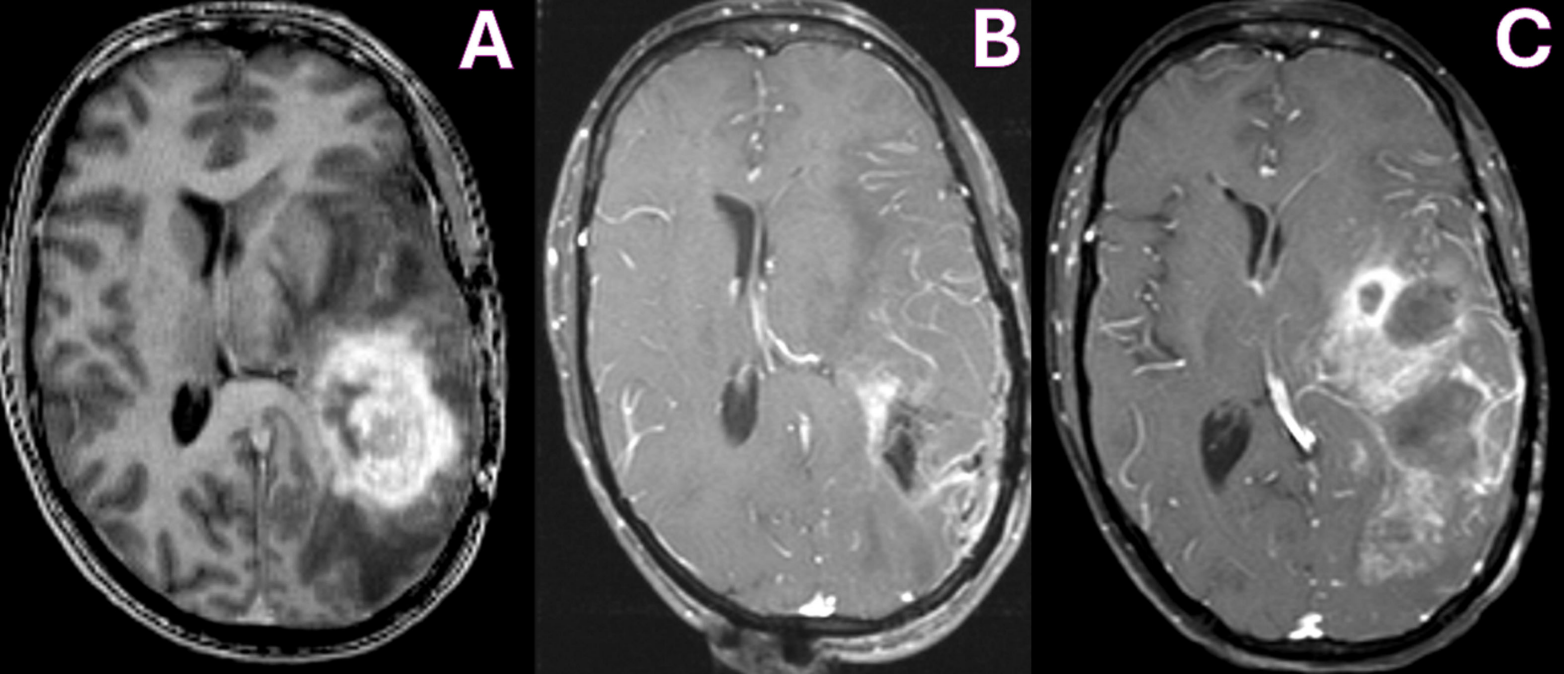
MRI T1-WI with contrast showing pre-operative tumor enhancement in the left temporoparietal lobe **(A)**, with improvement in enhancing tumor on immediate post-operative day 1 **(B)** and increased enhancement due to tumor recurrence at 4 months **(C)**.

In March 2011, she presented to our clinic presenting with language dysfunction, right-sided weakness (hemiparesis), and seizures. Her MRI revealed a large enhancing mass in the left temporoparietal lobe with significant vasogenic edema consistent with progressive disease (**Figure 1A**).

Because of her intermittent aphasia, the patient had limited understanding of her treatment options, but expressed desire to deliver her baby. Her parents fully understood the poor prognosis and also expressed their goal of protecting the unborn child. In addition to a comprehensive multidisciplinary tumor board discussion including neurosurgery, neuro-oncology, radiation-oncology, and OB-GYN, an ethics board was convened to weigh treatment options to optimize outcomes for the mother and fetus. Strategies to reduce mass effect and intracranial pressure to allow the patient to carry the fetus to age of pulmonary maturity included surgery alone or surgery followed by radiation and rechallenge with temozolomide or intralesional carmustine. The tumor board and ethics board felt that adjuvant radiation and systemic chemotherapy posed an increased risk to the fetus. The consensus decision was to proceed with the latter. The patient’s parents consented for her treatment.

She underwent surgery under general anesthesia. Anesthetics propofol and remifentanil were used for their favorable pharmacokinetic and physiologic profile in the gravid patient. Propofol was selected for its rapid induction and emergence, as well as its ability to reduce cerebral metabolic rate, cerebral blood flow, and intracranial pressure. Although remifentanil freely crosses the placenta, it was selected because of its short half-life and predictable metabolism, which would minimize fetal exposure. Intraoperative monitoring of fetal heart rate and uterine contractions was performed by an obstetric nurse with fetal ultrasound and cardiography. She underwent a successful left temporoparietal craniotomy and tumor resection, with implantation of six Gliadel wafers in the surgical bed (**Figure 1B**). Histopathology confirmed the diagnosis of a grade IV astrocytoma, IDH-1 mutant, ATRX loss, and MGMT unmethylated.

Postoperatively, her condition improved with only residual mild receptive aphasia, and she was able to ambulate without assistance. Upon discharge, she was placed under the care of her OB-GYN and continued a regimen of prednisone (15 mg oral twice daily), Decadron (2 mg oral twice daily), Keppra (1,500 mg oral twice daily), and Lamictal (100 mg daily).

Because of the pressing need for adjuvant therapy, a cesarean section (C-section) was performed in May 2011 at 26 weeks’ gestation after the fetus reached pulmonary maturity, resulting in the birth of a healthy baby girl. The patient’s newborn daughter was cared for in the Neonatal Intensive Care Unit and met appropriate milestones.

Two weeks after delivery, she presented with new neurologic symptoms including left eye ptosis and escalating headaches. Repeat magnetic resonance imaging (MRI) revealed increased contrast enhancement and vasogenic edema. She was initiated on Avastin therapy at a dose of 10 mg/kg every 2 weeks, resulting in subsidence of symptoms. She experienced delayed wound healing of her C-section wound following the second dose of Avastin, but her wound eventually healed.

In July 2011, 4 months after surgery and 2 months after initiation of Avastin therapy, she presented with worsening aphasia and weakness and had breakdown of her cranial wound. Her MRI revealed increased enhancement, acute hemorrhages in the frontal and temporal lobes, and obstructive hydrocephalus (**Figure 1C**). Given the poor prognosis, the patient was transitioned to hospice care. The baby girl remains healthy and is achieving developmental milestones beyond 14 years. Clinical time is outlined in **Table 1**.

**Table 1:** Clinical timeline of the case report.

Year	Event
2001	Initial diagnosis: Astrocytoma. Underwent first surgical resection.
2002	Recurrence with progression to anaplastic astrocytoma. Managed with repeat surgery → concurrent chemoradiation (temozolomide) → 9 cycles of adjuvant temozolomide.
March 2011	At 18 weeks’ gestation, presented with aphasia, right hemiparesis, and seizures. MRI: left temporoparietal mass with edema. Underwent craniotomy + implantation of six Gliadel wafers. Pathology: WHO grade IV astrocytoma.
Post-op 2011	Managed with prednisone, dexamethasone, Keppra, and Lamictal. Partial language dysfunction persisted but functional improvement noted.
May 2011	Cesarean section at 26 weeks for fetal lung maturity. Healthy female infant delivered; admitted to NICU and developed normally.
Late May 2011	Two weeks postpartum: recurrence of neurologic symptoms. MRI: increased enhancement/edema. Began Avastin (bevacizumab) 10 mg/kg q2w. Delayed wound healing noted but resolved.
July 2011	Four months post-surgery and 2 months into Avastin: worsened aphasia, weakness, cranial wound breakdown. MRI: acute frontal/temporal hemorrhages with obstructive hydrocephalus. Transitioned to hospice care.
>14 years later	Daughter remains healthy, meeting developmental milestones.

## Discussion

The management of malignant gliomas during pregnancy represents a medical and ethical dilemma that requires close collaboration between neurosurgery, oncology, and maternal–fetal medicine. Malignant gliomas are aggressive tumors with rapid progression and devastating neurologic consequences if untreated. Physiologic and hormonal changes during pregnancy may adversely influence tumor behavior. In a retrospective multi-institutional case series, Peeters et al. (3) report that 87% of patients who were diagnosed with tumors prior to pregnancy experienced an accelerated growth rate or volume of dynamic expansion (VDE) compared with pre-pregnancy rates (9.7 ± 14.5 mm/year vs. 1.0 ± 3.2 mm/year, *p* < 0.001). Furthermore, molecular signatures such as negative alpha-internexin and positive p53 were associated with a high risk for progression during pregnancy (3). Neurologic decline occurred in 38% of patients who demonstrated accelerated growth rates. Following delivery, the VDE decreased significantly in approximately 75% of cases. Conversely, for patients diagnosed during the second or third trimester, the growth rates did not significantly decrease after surgery, but only 25% demonstrated neurologic improvement. In addition to gestational age and neurologic function as determining factors for high-risk patients, Peeters et al. identified that molecular profiling may provide valuable prognostic information to identify patients at higher risk of progression during pregnancy and help tailor monitoring and management strategies (3).

In a limited case series, Tewari et al. identified eight women diagnosed with a malignant glioma during pregnancy, all of whom experienced a neurologic crisis (4). These findings highlight the urgency and complexity of managing such patients. Some consensus has emerged regarding the timing of treatment. Stable patients diagnosed in the first or early second trimester may benefit from waiting until the second trimester or after delivery for neurosurgery. In contrast, patients presenting in the late second or third trimester should defer these interventions, owing to elevated risks such as intracranial hemorrhage from increased maternal intravascular volume (4–7).

Our case represents a successful balance between maternal treatment and fetal safety in the context of rapidly advancing malignant glioma. Our decision to operate was guided by the principles of beneficence autonomy. Surgical cytoreduction reduced the tumor mass effect and intracranial pressure, thus stabilizing her neurologic status, prolonging her survival, and indirectly protecting fetal life by ensuring adequate uteroplacental perfusion. In regard to maternal autonomy, the patient had communicated her desire to prioritize the wellbeing of her unborn child prior to developing aphasia. Following surgical decompression, the patient’s neurologic symptoms improved, enabling continuation of pregnancy to the point of fetal viability. After a healthy infant was delivered by C-section, she was able to spend quality time bonding with her daughter.

Chemotherapy guidelines during pregnancy are less clear: first-trimester exposure is linked to severe teratogenic outcomes (6–9), while later exposures may cause low birth weight or neurobehavioral disorders (6–9). Recent evidence supports the cautious use of chemotherapy during pregnancy after the first trimester. A 2021 multicenter cohort study using the International Network on Cancer, Infertility and Pregnancy database found that chemotherapy after 12 weeks of gestation did not increase the risk of major congenital malformations compared to the general population (9), supporting its relative safety after the first trimester (6, 8, 9). Our case contributes to this growing body of evidence for chemotherapy use during pregnancy, specifically intralesional Gliadel.

Gliadel is approved by the Food and Drug Administration (FDA) for both recurrent and newly diagnosed high-grade gliomas. Intralesional applications bypass the systemic circulation and deliver high local concentrations of carmustine directly to the tumor (8, 9). Carmustine is a lipid-soluble, low-molecular-weight (0.214 kDa) molecule with potential for crossing the placenta. Animal studies using intraperitoneal carmustine at 1 mg/kg/day (about eight wafers’ worth) show embryotoxicity and teratogenicity in rats, but this delivery route and dosage far exceed clinical scenarios (9). Fleming and Saltzmann’s pharmacokinetic models showed steep drop-off curves with minimal effect to surrounding brain tissues. Systemic absorption of carmustine after placement reach peak plasma concentrations of only 10.2 ± 4.8 ng/mL around 3 h post-implantation, with roughly 80% bound to proteins, thus reducing its bioavailability (10). The drug’s short systemic half-life and its primary release within the first 5–7 days (but presence *in vivo* for 21 days) further limit fetal exposure (9, 11). The placental barrier expresses a variety of pumps and receptors that play a crucial role in maternal-to-fetal exclusion of chemotherapeutic substances (12).

In order to further investigate the effects of carmustine use during pregnancy, a review was conducted to identify all published case reports/studies that utilized carmustine during pregnancy. Only three other cases apart from this study were identified. In all three reported cases (**Table 2**), carmustine was administered intravenously during pregnancy, but always alongside other chemotherapeutic agents. Dipaola et al. combined carmustine with the Dartmouth regimen during the second trimester, resulting in the birth of a healthy child (13). In contrast, Li et al. reported complications in the infant following administration of the same regimen during the late first and second trimesters—likely attributable to exposure during the critical window of organogenesis (3–8 weeks), underscoring the traditional caution against first-trimester chemotherapy (14). Schapira et al. involved carmustine and procarbazine given before and during pregnancy, which resulted in a healthy child, offering a less confounded context to evaluate the safety of these agents (15). Our case is the first to use Gliadel wafers in isolation—a localized, polymer-based form of carmustine—administered alone during the second trimester. The child was born healthy and has met all developmental milestones beyond 14 years of age, suggesting that localized carmustine delivery may offer a safer alternative and warrants further study.

**Table 2:** Reported cases of carmustine use during pregnancy.

Study/Case report	Cancer type	Context	Carmustine used in isolation	Duration in pregnancy	Gestational age of delivery	Outcome
Dipaola et al. (13)	Metastatic melanoma	Carmustine used in combination with Dartmouth regimen	No	Second trimester	30 weeks	Healthy 1,520-g female neonate
Li et al. (14)	Hepatic metastasis (history of right eye melanoma)	Carmustine 100 mg/m² day 1 every other month with Dartmouth regimen	No	9th–21st week (late first trimester and second trimester)	34 weeks	Male neonate (2.75 kg) with normal Apgar. At 1 year: normal development but microphthalmos, small eyes, nystagmus, severe hyperopia, vision 20/400
Schapira et al. (15)	Diffuse histiocytic lymphoma	BCNU given with Procarbazine	No	5 months before conception and throughout the first and second trimesters	Not specified	A male infant who was phenotypically and genotypically normal was delivered
This case report	Glioblastoma multiforme	Left temporoparietal craniotomy with tumor resection and 6 Gliadel wafers placed	Yes (no chemo drugs given till delivery)	20th week (second trimester)	26 weeks, 4 days	Female neonate remains healthy and is achieving developmental milestones beyond 5 years

## Conclusion

In conclusion, the management of malignant glioma during pregnancy presents profound clinical and ethical challenges that demand a careful balance between maternal benefit and fetal safety. Our case adds to the limited but growing body of literature addressing this complex intersection, specifically describing the use of intralesional Gliadel chemotherapy during pregnancy. Pharmacokinetic data suggest that carmustine achieves maximal local concentration with minimal systemic absorption following local implantation, thereby reducing the likelihood of significant transplacental transfer. Although the theoretical fetal risks appear low and clinical adverse events remain rare, the absence of direct maternal and neonatal drug level measurements limits the definitive conclusions regarding safety. While further investigation is warranted, this case supports consideration of Gliadel wafers as an adjunctive option for managing malignant gliomas during pregnancy. Future studies should explore pharmacokinetic modeling, prospective safety data, and integration of emergent modalities such as focused ultrasound to enhance localized drug delivery across the blood–brain barrier (16).

## Data Availability

The raw data supporting the conclusions of this article will be made available by the authors, without undue reservation.
